# The AGPase Family Proteins in Banana: Genome-Wide Identification, Phylogeny, and Expression Analyses Reveal Their Involvement in the Development, Ripening, and Abiotic/Biotic Stress Responses

**DOI:** 10.3390/ijms18081581

**Published:** 2017-07-25

**Authors:** Hongxia Miao, Peiguang Sun, Qing Liu, Juhua Liu, Biyu Xu, Zhiqiang Jin

**Affiliations:** 1 Key Laboratory of Tropical Crop Biotechnology, Ministry of Agriculture, Institute of Tropical Bioscience and Biotechnology, Chinese Academy of Tropical Agricultural Sciences, Haikou 571101, China; miaohongxia@itbb.org.cn (H.M.); liujuhua@itbb.org.cn (J.L.); 2 Key Laboratory of Genetic Improvement of Bananas, Hainan Province, Haikou Experimental Station, Chinese Academy of Tropical Agricultural Sciences, Haikou 570102, China; sunpeiguang@catas.cn; 3 Commonwealth Scientific and Industrial Research Organization Agriculture and Food, Canberra, ACT 2601, Australia; Qing.Liu@csiro.au

**Keywords:** banana (*Musa acuminata* L.), AGPase, genome-wide identification, fruit development, abiotic stress, biotic stress

## Abstract

ADP-glucose pyrophosphorylase (AGPase) is the first rate-limiting enzyme in starch biosynthesis and plays crucial roles in multiple biological processes. Despite its importance, AGPase is poorly studied in starchy fruit crop banana (*Musa acuminata* L.). In this study, eight *MaAGPase* genes have been identified genome-wide in *M. acuminata*, which could be clustered into the large (APL) and small (APS) subunits. Comprehensive transcriptomic analysis revealed temporal and spatial expression variations of *MaAPLs* and *MaAPSs* and their differential responses to abiotic/biotic stresses in two banana genotypes, Fen Jiao (FJ) and BaXi Jiao (BX). *MaAPS1* showed generally high expression at various developmental and ripening stages and in response to abiotic/biotic stresses in both genotypes. *MaAPL-3* and *-2a* were specifically induced by abiotic stresses including cold, salt, and drought, as well as by fungal infection in FJ, but not in BX. The presence of hormone-related and stress-relevant *cis*-acting elements in the promoters of *MaAGPase* genes suggests that *MaAGPases* may play an important role in multiple biological processes. Taken together, this study provides new insights into the complex transcriptional regulation of *AGPases*, underlying their key roles in promoting starch biosynthesis and enhancing stress tolerance in banana.

## 1. Introduction

Starch is the most abundant storage polysaccharide in plants. As an important carbohydrate and energy source in the human diet and nutrition, starch is massively produced in cassava (*Manihot esculenta*) roots [1], potato (*Solanum tuberosum*) tubers [2], cereal seeds [3], and a number of fresh fruits such as banana (*Musa acuminata*) [4,5,6,7,8]. The biosynthesis of starch in plants is a complex process involving a variety of enzymes, such as ADP-glucose pyrophosphorylase (AGPase), granule-bound starch synthase (GBSS), soluble starch synthase (SS), starch branching enzyme (SBE), and debranching enzyme (DBE) [2,9,10]. AGPase, as the first key regulatory and rate-limiting enzyme in starch biosynthetic pathways, controls the starch content and affects the yield and quality of plants [2,9,10,11,12].

AGPase catalyzes the synthesis of the activated glycosyl donor and ADP-glucose from Glucose-1-phosphate (Glc-1-P) and ATP. It is a heterotetrameric enzyme that is composed of two large (APL) and two small (APS) subunits with typical NTP transferases domains (PF00483) [2,13]. The APL has a regulatory function with defective catalytic properties in *S. tuberosum*, Arabidopsis (*Arabidopsis thaliana*), and barley (*Hordeum vulgare*) [14]. In contrast, the APS is regulatory-deficient but it plays more of a catalytic role in plant tissues [15]. To date, genome-wide analysis has identified a variable number of *APL* and *APS* in numerous higher plants, including six in *A. thaliana* (four *APLs* and two *APSs*) [15], seven in rice (*Oryza sativa*) (four *APLs* and three *APSs*) [16,17], six in maize (*Zea mays*) (four *APLs* and two *APSs*) [13,18], six in sweet potato (*Ipomoea batatas*) (four *APLs* and two *APSs*) [19], four in sago palm (*Metroxylon sagu*) (three *APLs* and one *APS*) [20], four in kiwifruit (*Actinidia deliciosa*) (three *APLs* and one *APS*) [21], and four in tomato (*Solanum lycopersicon*) (three *APLs* and one *APS*) [22].

Further, recent biochemical and functional analyses have revealed that AGPase is playing an active role in multiple plant biological processes, including growth, development, environmental adaptation, and host-pathogen interactions. For example, in *A. thaliana*, *AtAGPase* was found to regulate plantlet growth rate and root development [23,24]. A tobacco (*Nicotiana tabacum*) *NtAGPase* was found to play a crucial role in petal expansion and growth [25]. The regulated expression of an *APS* derived from cotton (*Gossypium hirsutum*), together with the presence of starch in cotton fiber suggested an important role of *GhAGPase* in fiber development [26]. Temporally extended expression of *APL1* enhanced accumulation of starch and soluble solids in mature fruit in *S. lycopersicon* [22]. A mutant defective in *OsAPL4* showed impaired starch biosynthesis in pollen grains, which led to male sterility in *O. sativa* [27]. Furthermore, the expression of numerous *AGPase* genes in plants was found to be regulated by various stress conditions, including cold, salt, drought, and plant-pathogens [12,28,29,30]. In *S. tuberosum*, the expression of *AGPase* was regulated by low storage temperatures [30]. Under salt stress, *APL1* from *S. lycopersicum* was up-regulated in developing fruits, suggesting its role in the promotion of starch biosynthesis under the salinity stress in ABA- and osmotic stress-independent manners [28]. Under drought stress, AGPase activity was inhibited leading to significant losses in crop yield [12,31,32]. The Downy mildew (*Plasmopara viticola*) infection was shown to significantly induce AGPase activity and abnormal starch accumulation in grapevine leaves [29]. Taken together, these studies have revealed many important roles of the *AGPase* gene family in the regulation of plant growth, development, and responses to abiotic/biotic stresses.

With starch content reaching 74–88% of its dry weight in unripe fruit, *M. acuminata* is not only the most popular starch-rich fresh fruit in the world, but also an important staple food in some African and Latin American countries [6,7,8,33,34]. Starch biosynthesis is of crucial importance since starch content determines fruit yield, quality, and economic value of banana. As a large annual monocotyledonous herbaceous plant, banana is frequently affected or even destroyed by various abiotic/biotic stresses during growth and development. Genome-wide investigations of key genes involved in fruit development and abiotic/biotic stress responses have been conducted in several fruit crops, including banana [35,36,37]. The involvement of *AGPase* in the ripening of banana fruit has recently reported [5]. However, a genome-wide investigation of the *AGPase* gene family leading to an integrated picture of the potential functionality of this important rate-limiting enzyme involved in starch biosynthesis in banana is still lacking.

In this study, we identified eight *MaAGPase* genes from the entire banana genome and conducted a range of molecular analyses on their phylogenetic relationship, gene structure, protein motifs, spatial and temporal expression patterns, and expression in response to different abiotic/biotic stresses (cold, salt, drought, and fungal infection) in two banana varieties. In addition, we analyzed in silico the hormone-related and stress-relevant *cis*-elements in the promoters of *MaAGPase* genes. This comprehensive study serves to facilitate our understanding of *MaAGPase* in association with fruit development processes and abiotic/biotic stress responses, and provides a foundation for future studies of crop improvement involving *AGPases*. 

## 2. Results 

### 2.1. Identification and Phylogenetic Analysis of Banana MaAGPase Genes

DNA sequence database search using BLAST and the hidden Markov models (HMM) was conducted to identify all banana *MaAGPase* genes using the typical NTP transferase domains (PF00483), *A. thaliana AtAGPase* and *O. sativa OsAGPase* sequences as queries. A total of eight *MaAGPase* genes were identified in the *M. acuminata* genome and designated as *MaAPL-1a*, *-1b*, *-2a*, *-2b*, *-2c*, *-3*, *MaAPS-1*, and *-2* following the nomenclature of their respective orthologous genes in *A. thaliana*. The eight predicted MaAGPase proteins varied from 450 (MaAPS2) to 549 (MaAPL2a) amino acid residues in size, with relative molecular masses between 50.580 (MaAPS2) and 60.699 (MaAPL2a) kDa, and isoelectric points ranging from 6.53 to 8.91 (Figure S1 and Table S1).

**Figure 1:**
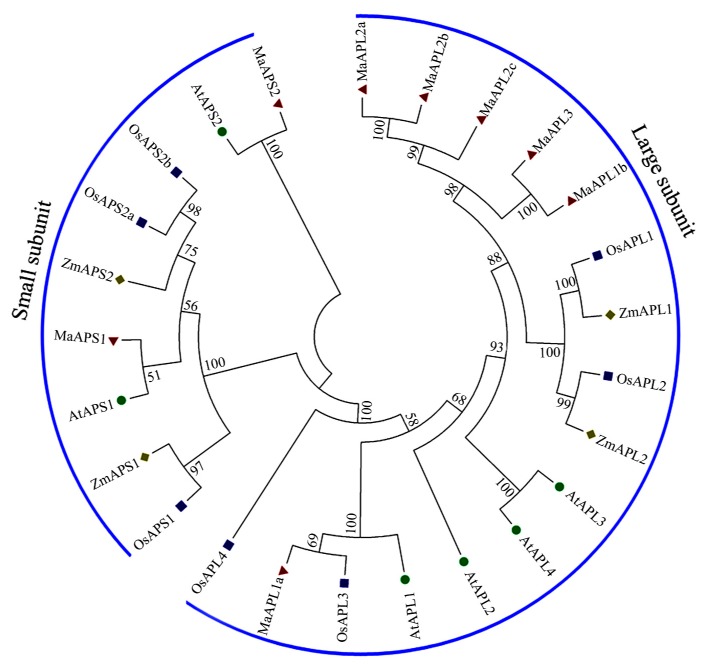
Phylogenetic analysis of the AGPases from Arabidopsis, rice, and banana. The Neighbor-joining tree was drawn using MEGA5.0 with 1000 bootstraps. Two subgroups were identified and classified as large subunit and small subunit. The circle, square, and triangle represent AGPase proteins from Arabidopsis, rice, and banana, respectively.

To study the evolutionary relationships between AGPase family proteins, a neighbor-joining tree was generated by aligning eight, six, and seven AGPase proteins from *M. acuminata*, *A. thaliana*, and *O. sativa*, respectively, using ClustalX and MEGA5.0 software. As shown in Figure 1, MaAGPase proteins were clearly grouped based on the size of their subunits. Six *M. acuminata APL* genes (*MaAPL-1a*, *-1b*, *-2a*, *-2b*, *-2c*, and *-3*) together with *A. thaliana AtAPL-1*, *-2*, *-3*, *-4*, and *O. sativa OsAPL-1*, *-2*, *-3*, *-4* were classified into the large subunit group, while the other two *MaAGPase* genes, i.e., *MaAPS-1* and *-2* together with *AtAPS-1*, *-2*, *OsAPS-1*, *-2a*, and *-2b*, were classified into the small subunit group.

### 2.2. Gene Structure and Conserved Motif Analysis of Banana MaAGPase Genes

**Figure 2:**
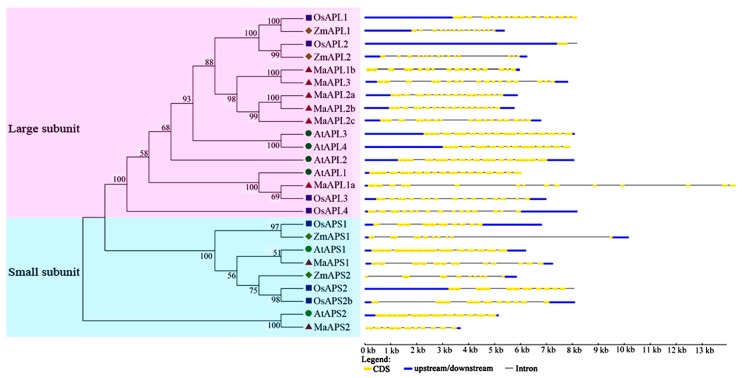
Gene structure analyses of *MaAGPases*. Exon-intron structure analyses were performed using the Gene Structure Display Server database. Blue boxes indicate upstream/downstream; yellow boxes indicate exons; black lines indicate introns. The circle, square, diamond, and triangle represent AGPase proteins from Arabidopsis, rice, maize, and banana, respectively.

Evolutionary analysis was further supported by the exon-intron structural divergence within families (Figure 2)*.* The gene organization and structural diversity of *MaAGPase* genes were analyzed by comparing to their orthologous genes derived from *A. thaliana*, *O. sativa*, and *Z. mays* using Gene Structure Display Server (GSDS) web based software. The gene organization was different between *APL* and *APS* genes in all the four species, indicating the diversity of expansion and evolution between these two subunit groups in plant *AGPase* genes (Figure 2).

**Figure 3:**
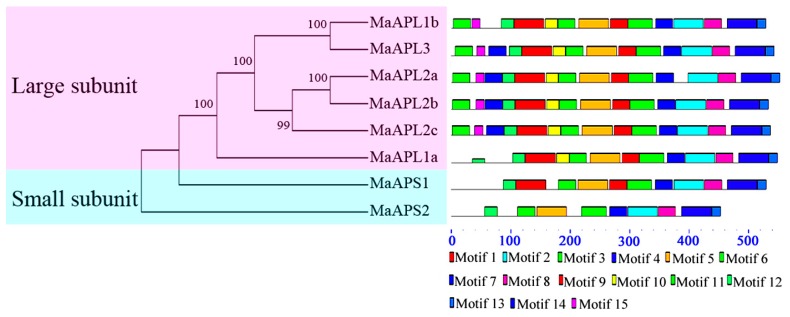
Phylogenetic and motif analyses of MaAGPase proteins. All proteins were identified by MEME database with the complete amino acid sequences of each MaAGPase identified. MaAGPases were classified into large and small subunits based on their phylogenetic relationship.

To further explore the protein structural diversity and predict the functionality of *MaAGPase* gene family, a total of 15 conserved motifs in MaAGPase proteins were identified using MEME5.2 software and annotated with the InterPro database (Figure 3, Table S2). Four motifs (motifs 1, 5–7) were annotated as NTP transferases (PF00483), which is characteristic of the AGPase protein family. In specific, six MaAPL proteins contain the motifs 1–15 whereas all MaAPS proteins contain motifs 2–8, 12, and 13. It is probable that the motif structure conservation and variation reflect the specific evolutionary history and functionality divergence among the eight MaAGPases.

### 2.3. Spatial Expression Analysis of MaAGPase Genes in Banana 

**Figure 4:**
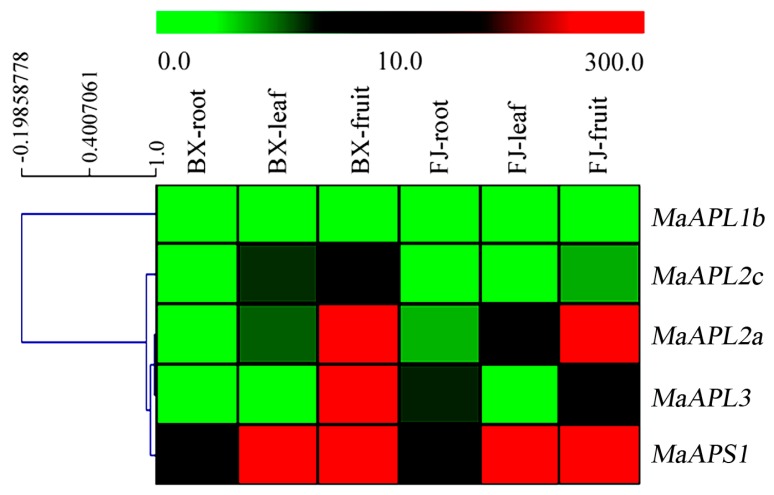
Expression of *MaAGPases* in roots, leaves, and fruits of BX and FJ banana varieties. The heat map with clustering was created based on the FPKM value of the *MaAGPases*. Differences in gene expression changes are shown in color in the green-red scale.

To investigate the spatial expression patterns of *MaAGPase* genes and their potential functional roles in banana growth and development, roots, leaves, and fruits were sampled from both BX and FJ genotypes and subjected to transcriptional analysis. Five *MaAGPase* genes were found to express in at least one of the tested tissues in both genotypes (Figure 4, Table S3). However, the expression of *MaAPL-1a*, *-2b*, and *MaAPS2* was not detected.

In BX, *MaAPS1* showed transcriptional expression in all tissue types examined. However, its highest expression was detected in the fruits with FPKM (fragments per kilobase of exon per million fragments, FPKM) > 10, together with other *MaAGPase* members including *MaAPL-2a*, *-2c*, and *-3*. 

In FJ, five, four, and five *MaAGPases* were expressed in roots, leaves, and fruit, respectively. *MaAPS1* was highly expressed in all the tissues examined (FRKM > 10). High expression levels of *MaAPL-2a* and *-3* were also detected in fruits.

In the three tissues examined, consistent spatial expression patterns of *MaAPL-1b*, *-2a*, *-3*, and *MaAPS1* were observed between the two genotypes, BX and FJ. *MaAPS1* exhibited consistently high level of expression (FRKM > 40) in all tissues examined in both genotypes, suggesting its important and divergent functional role in banana growth and development. This is in sharp contrast to the expression of *MaAPL2c* in fruit, which showed high level expression (FRKM > 47) in BX, but low expression (FRKM < 4) in FJ. This may suggest a differential functional role of this gene during fruit development in divergent banana cultivars. Taken together, this genotype-based spatial expression analysis warrants further study on the functional roles of *MaAGPase* gene family in banana.

### 2.4. Temporal Expression Analysis of MaAGPase Genes during Banana Fruit Development and Ripening 

**Figure 5:**
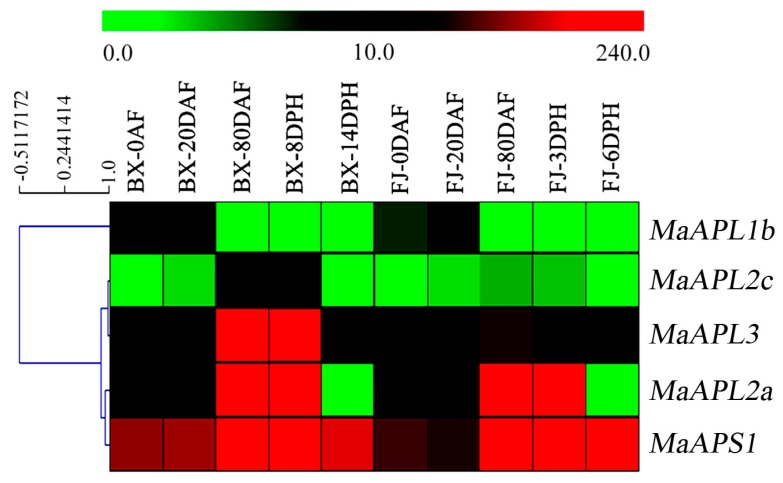
Expression of *MaAGPases* during different stages of fruit development and ripening in two banana varieties, BX and FJ. The heat map with clustering was created based on the FPKM value of the *MaAGPases*. Differences in gene expression changes are shown in color in the green-red scale.

To investigate the potential functionality of *MaAGPase* genes during banana fruit development and postharvest ripening, the expression of *MaAGPases* was analyzed in fruits sampled 0, 20, and 80 day after emergence from pseudostem (DAF) in both BX and FJ genotypes, 8 and 14 day after harvest (DPH) from BX, 3 and 6 DPH from FJ (Figure 5, Table S4). Except *MaAPL-1a*, *-2b*, and *MaAPS2*, five *MaAGPase* genes were expressed during fruit development and postharvest ripening in these two genotypes.

In BX, notably, *MaAPS1* showed the highest transcriptional accumulation (FRKM > 126) at all the examined stages of fruit development and ripening. Four other *MaAGPase* genes, including *MaAPL-1b*, *-2a*, *-2c*, and *-3* were also expressed at all the stages examined, but at relatively lower level than *MaAPS1*. Further, three (*MaAPL-1b*, *-2a*, and *-3*), three (*MaAPL-1b*, *-2a*, and *-3*), three (*MaAPL-2a*, *-2c*, and *-3*), three (*MaAPL-2a*, *-2c*, and *-3*) *MaAGPase* genes were detected in high transcriptional abundance (FRKM > 10) at 0, 20, 80 DAF, and 8 DPH, respectively. Similar to BX, *MaAPS1* exhibited high expression (FPKM > 76) at all the examined stages of fruit development and ripening in FJ. Four other *MaAGPase* genes, including *MaAPL-1b*, *-2a*, *-2c*, and *-3* were also expressed, among which two (*MaAPL-2a* and *-3*), three (*MaAPL-1b*, *-2a*, and *-3*), two (*MaAPL-2a* and *-3*), two (*MaAPL-2a* and *-3*), and one (*MaAPL3*) genes showed high transcription level (FPKM > 10) at 0, 20, 80 DAF, and 3 and 6 DPH, respectively. During fruit development and ripening, the two large subunit genes (*MaAPL-3* and *-2a*) showed similar expression patterns in both BX and FJ. In contrast, another large subunit gene, *MaAPL2c*, showed much higher expression level (FPKM > 29) in BX, compared to FJ (FPKM < 3.9) at 80 DAF and 8 DPH. These findings imply a significant transcriptional response of *MaAPL2c* during fruit development and early-stage ripening processes in BX. In addition, *MaAPS1* displayed high transcriptional abundance (FPKM > 76) at all phases in both BX and FJ, implying the high expression of small subunit gene *MaAPS1* could be important in the regulation of fruit development and ripening.

### 2.5. Expression of MaAGPases under Cold, Salt, and Osmotic Stresses

**Figure 6:**
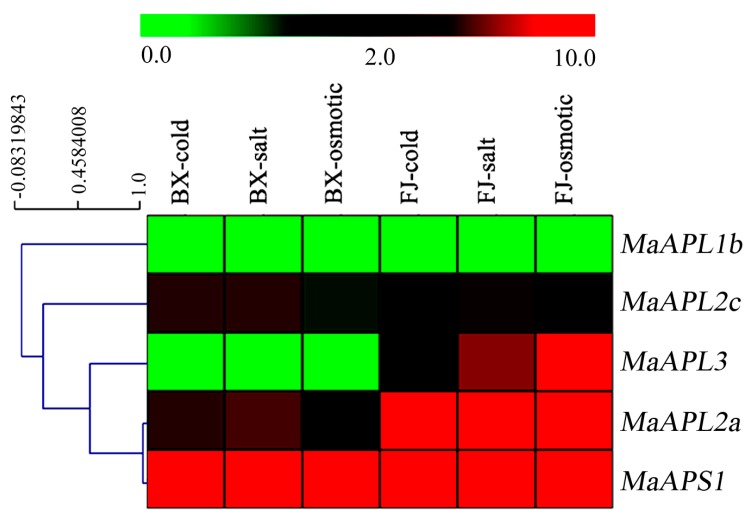
Expression of *MaAGPases* in response to cold, salt, and osmotic stresses in two banana varieties, BX and FJ. The heat map with clustering was created based on the FPKM value of the *MaAGPases*. Differences in gene expression are shown in color in the green-red scale.

*MaAGPase* expression in response to cold, salt, and osmotic treatments was analyzed by transcriptome data in BX and FJ. Five out of the eight *MaAGPase* genes showed variable levels of transcription in banana leaves following the abiotic stress treatments (Figure 6, Table S5). 

In BX, the expression of *MaAPS1* was strongly up-regulated (FPKM > 134) by all the three abiotic stress treatments. The two large subunit genes, *MaAPL-2a* and *-2c* were only moderately up-regulated by cold and salt treatments (FPKM > 2.0). A similar level of up-regulation by osmotic treatment was also observed in *MaAPL2a*, but not in *MaAPL-2c*. *MaAPL-1b* and *-3* were all down-regulated by any of the three abiotic treatments (FPKM < 0.5). *MaAPL2c* was also down-regulated by osmotic treatment, but not by cold and salt treatments. Similar to BX, the expression of *MaAPS1* was significantly induced (FPKM > 329) in FJ by cold, salt, and osmotic treatments. In FJ, moderate inductions were also observed in the three large subunit genes, including *MaAPL-2a*, *-2c*, and *-3* (FPKM > 2.5) under each of the three abiotic stress treatments. The expression of *MaAPL1b* in FJ was down-regulated (FPKM < 0.06) following each of the three abiotic stress treatments, consistent with the observation in BX. *MaAPL3*, was specifically up-regulated (FPKM > 2.6) in FJ, but maintained low expression in BX (FPKM < 0.19) under either of the three stress treatments, implying a significant genotypic variation in response to abiotic treatments.

### 2.6. Expression Profiles of Banana MaAGPases in Response to Fusarium Oxysporum f.sp. Cubense (Foc) Tropical Race 4 (TR4) Infection

**Figure 7:**
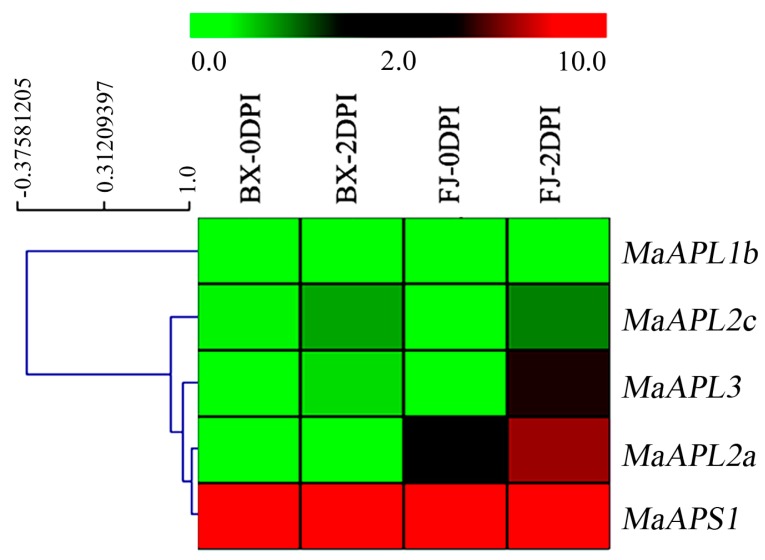
Expression of *MaAGPases* in response to fungal disease in two banana varieties, BX and FJ. The heat map with clustering was created based on the FPKM value of the *MaAGPases*. Differences in gene expression changes are shown in color in green-red scale.

To investigate the potential functional roles of *MaAGPase* genes in defense against fungal diseases in banana, *MaAGPase* gene expression was analyzed in the entire root system of BX and FJ plants following 0 and 2 days post-infection (DPI) with the Panama disease, Foc TR4. Five out of the eight *MaAGPase* genes showed transcriptional changes in response to the fungal infection in both BX and FJ genotypes (Figure 7 and Table S6).

In both BX and FJ, five *MaAGPase* genes, including *MaAPL-1b*, *-2a*, *-2c*, *-3*, and *MaAPS1*, were expressed in Foc TR4 infected roots. In BX, *MaAPS1* exhibited a 1.6-fold increase in sequence abundance at 2 DPI. In FJ, *MaAPL-2a*, *-3*, and *MaAPS1* were all up-regulated, with 46-, 3.5-, and 1.6-fold increase, respectively.

### 2.7. Validation of the Differentially Expressed MaAGPase Genes by Quantitative Real-Time Polymerase Chain Reaction (qRT-PCR) Analysis

**Figure 8:**
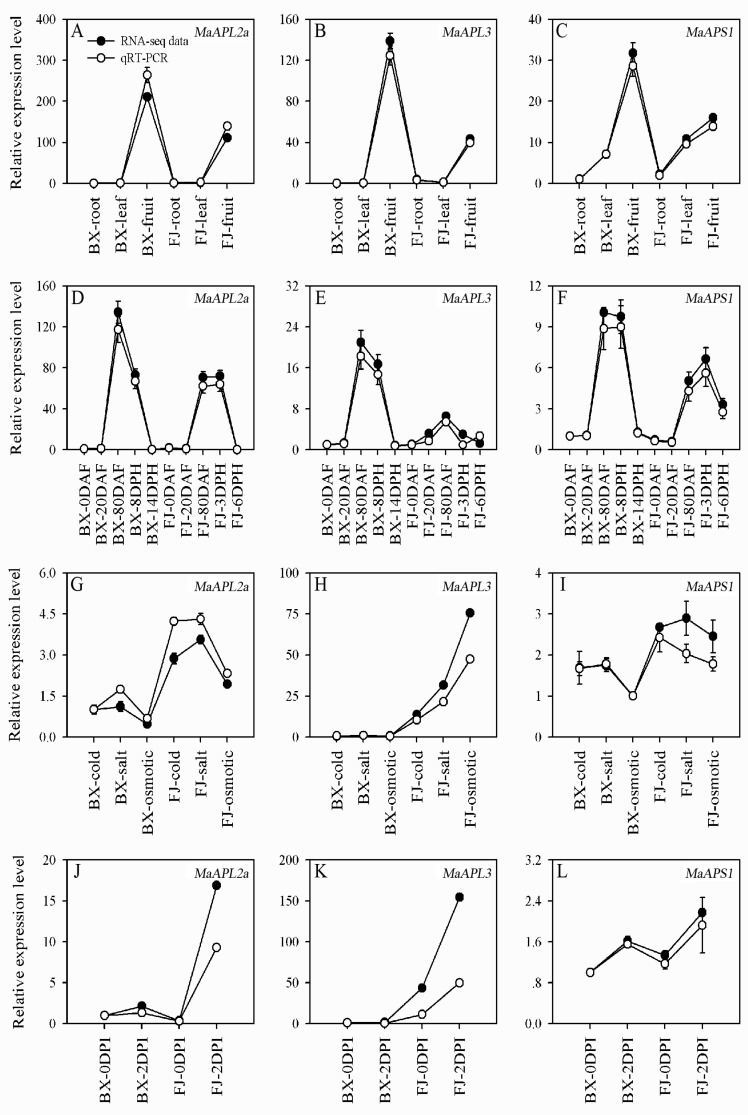
Relative expression of *MaAGPases* in two banana varieties, BX and FJ, by qRT-PCR. (**A**–**C**) Expression of *MaAPL-2a*, *-3*, and *MaAPS1* in different organs. (**D**–**F**) Expression of *MaAPL-2a*, *-3*, and *MaAPS1* at different stages of fruit development and ripening. (**G**–**I**) Expression of *MaAPL-2a*, *-3*, and *MaAPS1* in response to cold, salt, and osmotic stresses. (**J**–**L**) Expression of *MaAPL-2a*, *-3*, and *MaAPS1* in response to fungal infection. Data are presented as means ± standard deviations of *n* = 3 biological replicates.

RNA-seq analysis indicated that *MaAPL-2a*, *-3*, and *MaAPS1* had exhibited high levels of expression in different tissues, or during most stages of fruit development and ripening, or being induced by abiotic/biotic stress treatments in BX or FJ. Such a feature of these three genes was verified by qRT-PCR analysis. After normalization, all the examined *MaAGPases* with the exception of *MaAPL3* in FJ at 6DPH and *MaAPS1* in BX at 8DPH showed consistent expression patterns with the RNA-seq analysis (Figure 8). The correlation coefficient between RNA-seq and qRT-PCR data ranged from 0.8750 to 0.9999 (Table S7). These results indicate that RNA-seq analysis supplied suitable expression results for both banana varieties. 

### 2.8. In Silico Identification of Hormone-Related and Stress-Related Cis-Acting Elements in the Promoters of MaAGPase Genes

Promoter is a molecular switch that initiates gene expression and studies of the *cis*-acting elements in promoter sequence have been used as a useful tool for investigating regulatory mechanism of gene expression and gene function [10]. As shown in Table 1, five hormone-related (ABA, auxin, MeJA, ethylene, and gibberellin) and seven abiotic or biotic stress-related (anaerobic, fungal, heat, cold, drought, salicylic acid, and defense) elements were identified in the promoters of these *MaAGPase* genes. No less than two hormone- or stress-related elements were found in each of the eight *MaAGPase* promoters, suggesting the potentially important roles of *AGPases* in the regulation of development, ripening, and stress responses through the hormone- and stress-related *cis*-acting elements.

## 3. Discussion

In spite of the economic and social importance of bananas, research on banana plants has generally been slow relative to other crops, especially with respect to fruit development and responsiveness to abiotic/biotic stresses [8]. AGPase is the first rate-limiting enzyme in starch biosynthetic pathways and it has also been reported to play crucial roles in the regulation of plant growth, development, and in response to environmental stresses in many plant species [12,23,29,30]. Based on genome-wide search in *M. acuminata*, we have identified eight *MaAGPase* genes, which could be classified into two distinct groups based on the size of their subunit and phylogenetic relationship. Such a finding is consistent with the classification of *AGPases* in other higher plants as represented in *A. thaliana* and *O. sativa* [15,16]. The phylogenetic analyses were further supported by gene structure and conserved motif analyses. The large and small subunits in *MaAGPases* are distinct in exon-intron organization. It was found that the large subunits harbor 14–15 exons, whereas small subunits contain significantly fewer, only 9–10 exons. Such a structural feature of *MaAGPases* has also been observed in other plant species, such as *Triticum aestivum* [38], *I. batatas* [39], and *H. vulgare* [40]. Moreover, these two types of subunits also differ in their conserved motifs (Figure 3), as previously observed in *T. aestivum* [10]. 

The *AGPase* family has been reported to participate in the fruit development and ripening process in many plant species, such as *S. lycopersicum* [22], *T. aestivum* [41], and *Vitis vinifera* [29]. In *S. lycopersicum*, *APL1* was found highly expressed in developing fruits, implying regulatory control of AGPase activity and heterotetramer stability [22]. In *T. aestivum*, *AGPase* expression was closely related to starch synthesis and degradation in pericarp during caryopsis development [41]. In the current study, we found that more than 63% *MaAGPases* were expressed during developmental and ripening stages in both BX and FJ varieties. More than 25% of the expressed *AGPase* genes were highly expressed (FPKM > 10) (Figure 5), implying that *MaAGPases* may be extensively involved in the fruit development and ripening in banana. In this study, we have also found that both *MaAPL-3* and *-2a* showed specifically high levels of expression during fruit development stage and early-stage ripening, in contrast to *MaAPS1* which displayed constantly high expression at all phases of fruit development and ripening. Interestingly, *MaAGPase* expression patterns at a certain stage varied greatly between genotypes, for instance, the *MaAPL2c* was expressed more than ten-fold higher in BX than FJ at the late stage of banana fruit development. The BX genotype, with AAA genome, is known to produce high yield and high quality fruits with long fingers and extended shelf life compared to the FJ genotype with AAB genome [35]. It is tempting to assume that the increase of *MaAGPase* expression during fruit development augments starch synthesis ability, thereby enhancing the quality and yield of banana fruit. Such a finding in this study is consistent with previous studies suggesting that the A-genome harbors more genes that are important for banana yield and quality and could be used as a target in breeding programs [8,35]. Bananas are extremely sensitive to abiotic stresses, and can suffer heavy yield and quality penalties at cold, salt, or drought stress conditions [35]. In the present study, 63% *MaAGPases* showed transcriptional changes following abiotic stress treatments, including cold, salt, and osmotic stresses (Figure 6). In this report, for the first time in banana, we have demonstrated that *MaAGPase* genes exhibit extensive and diverse responses to abiotic stresses. This is consistent to previously reported induction of *AGPase* expression by cold, salt, and drought stresses in other plants, such as *S. tuberosum* [30], *S. lycopersicum* [28], and *T. aestivum* [31]. Of particular interest, comparative transcriptome analysis clearly demonstrated that higher number of *MaAGPase* genes were significantly up-regulated in FJ than in BX when subjected to cold, salt, and drought treatments (Figure 6). It has been reported that B-genome-containing banana varieties are more resilient to abiotic stresses [42,43]. FJ, with its genome constitution as AAB, has been reported to have more tolerance to abiotic stress in comparison to the banana genotypes containing only A-genome [35]. 

Banana production could be devastated by fungal disease caused by Foc TR4 [44]. In grapevine, the *P. viticola* infection was found to directly increase AGPase activity [29]. Similarly, up-regulation of *AGPase* in leaves leading to the enhanced starch biosynthesis as the result of Huanglongbing (HLB) infection has also been reported in citrus species [45]. In addition, depending on the pathosystems, an alteration in starch content in plant tissues was found to be related to the interactions between host plant and biotrophic pathogens [46]. In the current study, the transcriptional up-regulation of *MaAGPase* genes in response to Foc TR4 infection may suggest their possible role in response to fungal infections in banana. Furthermore, the fact that more *MaAGPase* genes were found in response to Foc TR4 infection in FJ than BX may suggest the possible involvement of B-genome in protection against biotic stress in banana. 

In wheat, the first 400 bp of the 5′ region along with the first intron of 88 bps in *APL* was found to be important for gene expression and starch accumulation in endosperm [47]. More recently, several *cis*-acting regulatory elements have been identified in the *AGPase* promoter, which are involved in the spatial and temporal gene expression and in response to abiotic stresses [10,47,48,49]. In this study, for the first time in banana, we have identified five hormone-related and seven stress-relevant *cis*-regulatory elements in the promoters of eight *MaAGPase* genes. Such a finding supports the genotype-wide transcriptome analysis of *AGPase* genes, which are likely playing an important role in the regulation of multiple development processes and in response to abiotic/biotic stresses in banana. 

## 4. Materials and Methods

### 4.1. Plant Materials

Two banana cultivars, BaXi Jiao (*M. acuminata* AAA group cv. Cavendish, abbreviated as BX) and Fen Jiao (*M.* AAB group Fenjiao, abbreviated as FJ), were obtained from the banana plantation at the Chinese Academy of Tropical Agricultural Sciences (Danzhou, Hainan, China), and were selected to perform comparative analyses because of their distinct characteristics. BX is a triploid (AAA) cultivar featured with high yield, high quality, and long-term storage. FJ is also a triploid cultivar, but with a different genotype (AAB), featured by good flavor, rapid ripening, and tolerance to abiotic stresses. Root, leaf, and fruit at 80 DAF were selected for spatial *AGPase* expression analysis. For temporal expression analysis, developing banana fruits of 0, 20, and 80 DAF, representing fruit developmental stages of budding, cutting flower, and harvest stages, respectively, were collected from both banana varieties. BX fruits stored for 0, 8, and 14 DPH and FJ fruits at 0, 3, and 6 DPH, representing the three progressive ripening stages based on color of the fruit, including green, yellowish green, and yellow, respectively, have been selected for post-harvest analysis. For salt and osmotic treatments, five-leaf stage banana plants (grown at 28 °C, 70% relative humidity, 200 μmol·m^−2^·s^−1^ light intensity, and 16 h light/8 h dark cycle) were irrigated with 300 mmol·L^−1^ NaCl and 200 mmol·L^−1^ mannitol for 7 days. For cold treatment, banana plants were maintained at 4 °C for 22 h prior to analysis. For Foc TR4 treatments, roots of five-leaf stage banana seedlings were dipped in a Foc TR4 spore suspension of 1.5 × 10^6^ condia/mL. The entire root system was harvested at 0 and 2 DPI, immediately frozen in liquid nitrogen, and stored at −80 °C until expression analysis.

### 4.2. Identification and Phylogenetic Analyses of MaAGPase Proteins in Banana

Banana AGPase proteins were downloaded from the DH-Pahang (*M. acuminata*, A-genome, 2*n* = 22) genome database (Available online: http://banana-genome.cirad.fr) [34]. AGPase amino acid sequences from *A. thaliana* and *O. sativa* were obtained from the TAIR (Available online: http://www.arabidopsis.org) and RGAP (Available online: http://rice.plantbiology.msu.edu) databases, respectively. The typically conserved NTP transferase domains (PF00483, Available online: http://pfam.sanger.ac.uk) in AGPase were used to query the predicted banana AGPase proteins using HMMER software (Available online: http://hmmer.org). BLAST analysis was also used to identify the predicted banana AGPases with all the AGPases from *A. thaliana* and *O. sativa* queries. The conserved domain search of the potential banana AGPases was further validated using conserved domain database (CDD) (Available online: http://www.ncbi.nlm.nih.gov/cdd) and PFAM (Available online: http://pfam.sanger.ac.uk) databases. The accession number of all identified banana AGPases were added in Table S1. A bootstrap neighbor-joining phylogenetic tree with the deduced AGPase amino acids sequences from *M. acuminata*, *A. thaliana*, and *O. sativa* were constructed by Clustal X 2.0 and MEGA 5.0 software with bootstrap values for 1000 replicates [50].

### 4.3. Characterization Analysis of Protein Properties and Gene Structure

Molecular mass and isoelectric points of the MaAGPase proteins were predicted by the ExPASy database (Available online: http://expasy.org/). MaAGPase protein motifs were analyzed with MEME software (Available online: http://meme-suite.org) and annotated by searching InterProScan database (Available online: http://www.ebi.ac.uk/Tools/pfa/iprscan). Gene structure features of *MaAGPases* were identified using GSDS software (Available online: http://gsds.cbi.pku.edu.cn) by comparing genomic sequences and predicted coding regions. Promoter sequences of *MaAGPase* genes were obtained from the banana genome database (Available online: http://banana-genome.cirad.fr). A fragment of 2000 bp upstream sequences derived from each *MaAGPase* gene was used to predict the transcription start site using database (Available online: http://www.fruitfly.org/seq_tools/promoter.html) and the *cis*-acting elements via PlantCARE software (Available online: http://bioinformatics.psb.ugent.be/webtools/plantcare/html). 

### 4.4. Transcriptomic Analysis

Transcript accumulation of *MaAGPase* genes in different organs, at different developmental and ripening stages, and under different abiotic/biotic stresses was investigated in the BX and FJ genotypes. Roots, leaves, and fruits at 80 DAF were used for the expression analysis of *MaAGPase* genes in different tissues. Pulps at 0, 20, and 80 DAF were collected for the expression analysis of *MaAGPase* genes during fruit development. Pulps at 8 and 14 DPH in BX and at 3 and 6 DPH in FJ were collected for expression analysis of *MaAGPase* genes during fruit postharvest ripening. The leaves derived from banana seedlings at five-leaf stage treated with 200 mM mannitol for 7 days, 300 mM NaCl for 7 days, or low temperature (4 °C), or Foc TR4 treatments were collected to expression analysis of *MaAGPase* genes under different abiotic/biotic stresses. All samples were collected and frozen quickly in liquid nitrogen and stored at −80 °C for RNA extraction. Total RNA was extracted using the plant RNAprep pure Kit (TIANGEN Biotech, Beijing, China) and used to construct cDNA libraries following manufacturer’s instruction. Deep sequencing was performed with the GAII kit (Illumina, San Diego, CA, USA) following manufacturer’s instructions with two technical replicates per sample. The sequencing depth was 5.34× on average. Adapter sequences in the raw sequence reads and low quality sequences were removed using FASTX-toolkit and FastQC, respectively, and clean reads were obtained and mapped to the DH-Pahang genome (*M. acuminata*, *2n* = 22, A genome). Transcriptome assemblies were constructed with Cufflinks [35]. Gene expression levels were calculated as FPKM. DEGseq was used to identify differentially expressed genes.

### 4.5. QRT-PCR Analysis 

Expression levels of *MaAGPases* in different tissues, different stages of fruit development and ripening, and in response to cold, salt, osmotic, and Foc TR4 stresses were further analyzed by qRT-PCR analysis using SYBR^®^ Premix *Ex* Taq^TM^ kit (TaKaRa, Shiga, Japan) chemistry on a Stratagene Mx3000P detection system (Stratagene, San Diego, CA). Primer pairs that had high specificity and efficiency according to melting curve analysis and agarose gel electrophoresis were selected to conduct quantification analysis (Table S8). Amplification efficiencies of primer pairs ranged from 0.9 to 1.1. The banana *MaActin* (EF672732) and *MaUBQ2* (HQ853254) were selected as internal controls to normalize the relative expression levels of *MaAGPase* genes. The relative expression levels of *MaAGPases* were assessed based on 2^−ΔΔ*C*t^ method [51]. Each sample contains three replicates.

### 4.6. Statistical Analysis 

Three biological replicates were performed for each sample. Statistical analyses were performed using Microsoft Excel and SPSS (Chicago, IL). Analysis of variance was used to compare the statistical difference based on Student’s *t*-tests, at significant levels of *p* < 0.05 (*) and *p* < 0.01 (**).

## 5. Conclusions

We identified eight *MaAGPase* genes from the banana genome, which could be classified into APL and APS, representing the large and small subunits respectively, following phylogenetic analysis, gene structure, and conserved protein motif analyses. Spatial and temporal expression profiles of *MaAGPases* in two triploid banana varieties revealed that *MaAPLs* and *MaAPSs* are differentially expressed during fruit development and ripening, suggesting distinct functions during the fruit development and ripening. The expression patterns of *MaAGPases* in response to abiotic/biotic stresses may shed light on their possible involvements in stress signaling pathway regulation, especially in the B-genome-containing banana varieties. Furthermore, the identification of hormone-related and stress-related *cis*-acting elements in *MaAGPase* promoters provides further evidence for their active role in response to fruit development, ripening, and abiotic/biotic stress signaling. These results will advance our understanding of the functional characterization of *AGPase* genes in banana, and provide a solid foundation for further genetic improvement of banana fruit quality and resistance to various environmental stresses.

## Figures and Tables

**Table 1:** Kinds and numbers of the known hormone-related and stress-related elements found in the upstream regions of *MaAGPase* genes.

Element	*ABRE* (ABA)	*ARE* (Anaerobic)	AuxRR(Auxin)	Box-W1 (Fungal)	CGTCA-Motif (MeJA)	Circadian	*ERE* (Ethylene)	*GARE* (Gibberellin)	*HSE* (Heat)	*LTR* (Cold)	*MBS* (Drought)	TCA-Element (Salicylic Acid)	TC-Rich Repeats (Defense)	Total
*MaAPL1a*	0	2	0	0	0	1	0	0	0	0	4	0	0	7
*MaAPL1b*	0	2	0	1	3	1	0	0	0	0	1	0	2	10
*MaAPL2a*	3	0	1	1	5	2	0	0	0	0	1	0	3	16
*MaAPL2b*	3	0	1	1	4	2	0	0	0	0	1	2	3	17
*MaAPL2c*	4	1	0	0	1	0	1	1	0	2	0	1	1	12
*MaAPL3*	4	0	1	0	7	1	0	1	0	0	1	0	0	15
*MaAPS1*	2	0	0	0	0	1	0	1	0	0	3	0	0	6
*MaAPS2*	0	0	0	0	0	0	0	0	2	0	0	0	0	2

*ABRE*, ABA responsive element; *ARE*, anaerobic responsive element; AuxRR, auxin responsive element; Box-W1, fungal elicitor responsive element; CGTCA-motif, MeJA responsive element; Circadian, circadian control; *ERE*, ethylene responsive element; *GARE*, gibberellins responsive element; *HSE*, heat stress responsive element; *LTR*, low temperature responsive element; *MBS*, MYB binding site involved in drought induction; TCA-element, salicylic acid responsive element; TC-rich repeats, defense responsive elements.

## Supplementary Materials

Supplementary materials can be found at www.mdpi.com/1422-0067/18/8/1581/s1.
