# Supplementary material for: The AGPase Family Proteins in Banana: Genome-Wide Identification, Phylogeny, and Expression Analyses Reveal Their Involvement in the Development, Ripening, and Abiotic/Biotic Stress Responses
Source: Int J Mol Sci. 2017 Jul 25;18(8):1581. doi: 10.3390/ijms18081581 (PMC5577994; doi:10.3390/ijms18081581)
Supplement: Supplementary file 1 [file ijms-18-01581-s001.zip › Figure S1.pdf]

| | |
|---|---|
| MaAPL1a | MSFSHSPTLPLLRQGDMVMCMNSRMVPLATVGRLPGRPIPRLVGTSRDGQTMGTRLDWQR |
| MaAPL1b | ----------MMDASSVGLKSSACLSQIKRGVLDAEES-----GFAVGSR----IMAWKP |
| MaAPL2a | -----------MEVCSVGMKANVCFGQVKRRSVGSGESGIWGEGIGGGGGL-KIKVWET |
| MaAPL2b | -----------MEVCSVGMKANVCFGQVKRRSVGSGESGIWGEGIGGGGGL-KIKVWET |
| MaAPL2c | -----------MDVCCVGSMANACFSQVKRASVGCRESGIWGDGIGNGPK---TTKVWDS |
| MaAPL3 | ------MIAPLMDVSSVGLKANACFSHVKRGVLGSGESGIWGDGSAGASR----IRAWES |
| MaAPS1 | -----MAAMGIAKLPPLNNSVHPAHRKSPTPFLASSDPRLSSSFHVSGGND--NILLLRK |
| MaAPS2 | --------------MVILRLAPSFSLVRRPTTVSMQHS----DLNTQRK------RWRQ |
| | . |
| MaAPL1a | YLRCLRSGRGSMVGAAEKRLVVQMAIVTTDVET------EVKFRDLDLERRNPGTVVAVI |
| MaAPL1b | KVVRSAKNCCCWVGEFKAGAGVFAITSAVNKET------VIQAPRFGKHKPDARSVASII |
| MaAPL2a | KVVKGVK-RRNSLG-----AAVAVLTSDVSEET-----MVLHAPMFGYRTAEPKSVASII |
| MaAPL2b | KVVKGVK-RRNSLG-----AAVAVLTSDVSEET-----MVLHAPMFGYRTAEPKSVASII |
| MaAPL2c | KVAKSLK-TRSSFGGRKTGVAFSVLTSDVDQET-----MVFHAPMFGRRTADPKSVASII |
| MaAPL3 | KVAKNVK-SGRWVGGFKAGVAFSDLTSDVNQETLINDPYVIQAPMFGKHKPDPKSVASII |
| MaAPS1 | AAIASGR--RTASG-----ARTPVLVSPKAVSD-------SRSSQTCLDPDASRSVLGII |
| MaAPS2 | PHVFLDT-------------MDTTLCQSHKDS-------------PLFPPLNQSVLAII |
| | .* .* |
| MaAPL1a | LGGGAGTRLFPLTKRRAKPAVPIGGAYRLIDVPMSNCINSGINKVYILTQFNSASLNRHL |
| MaAPL1b | LGGGAGAQLFPLTSTRATPAVPVGGCYRLIDIPMSNCINSGINKIFIMTQFNSASLNRHI |
| MaAPL2a | LGGGAGTQLFPLTSTRATPAVPIGGCYRLIDIPMSNCINSGINKIFIMTQFNSASLNRHI |
| MaAPL2b | LGGGAGTQLFPLTSTRATPAVPIGGCYRLIDIPMSNCINSGINKIFIMTQFNSASLNRHI |
| MaAPL2c | LGGGAGTQLVPLTSTRATPAVPIGGCYRLIDIPMSNCINSGINKIFIMTQFNSASLNRHI |
| MaAPL3 | LGGGPGAQLFPLTSTRATPAVPVGGCYKLIDIPMSNCINSGINKIFIMTQYNSASLNRHI |
| MaAPS1 | LGGGAGTRLYPLTKKRAKPAVPLGANYRLIDIPVSNCLNSNILKIYVLTQFNSASLNRHL |
| MaAPS2 | CDDEPGTKLYPLTKRRSKSAIPVSAHYRIIDFVLG------------------------ |
| | *..* *** *. *.*. *..** . |
| MaAPL1a | ARAYNFSNGVSFGDGFVEVLAATQTPGVEGKRWFQGTADAVRQFHWLFEDAKGKDIKDVL |
| MaAPL1b | YRTFNFGNGINFGDGFVEVLAATQSPGESGMNWFQGTADAVRQFTWVFEDNRNKNIQYIL |
| MaAPL2a | SRTYIFGNGINFGDGFVEVLAATQTPGEAGMNWFQGTADAVRQFTWVFEDNRNKNIEHIV |
| MaAPL2b | SRTYIFGNGINFGDGFVEVLAATQTPGEAGMNWFQGTADAVRQFTWVFEDNRNKNIEHIV |
| MaAPL2c | SRTYNFGNGINFGDGFVEVLAATQTPGEAGMNWFQGTADAVRQFTWVFEDNKNKNIEHIL |
| MaAPL3 | YRTFNFGNGINFGDGFVEVLAATQSPGEAGMNWFQGTADAVRQFIWVFEDNRNKNIDYIM |
| MaAPS1 | SRAYASNMGGYQNEGFVEVLAAQQSPENP--NWFQGTADAVRQYLWLFEEHN---VMEYL |
| MaAPS2 | ------------KDGFVEVLTTYQSAEDLS--WFKGNADAVRRWLWLLEEHQ---VKDFL |
| | .******.. *. **.*.*****. *. *. . . |
| MaAPL1a | ILSGDHLYRMDYMDFVQDHRQSGADITISCMPMDDSRASDFGLMKIDNKGRVISFSEKPK |
| MaAPL1b | ILSGDQLYRMDYMDFVQKHVDTGADITISCVPVGASRASDYGLIKIDKAGKIIQFSEKPK |
| MaAPL2a | ILSGDQLYRMDYMDLVQKHIDTGADITVSCVPVGHSRASDYGLVKIDKTGRIFQFSEKPN |
| MaAPL2b | ILSGDQLYRMDYMDLVQKHIDTGADITVSCVPVGHSRASDYGLVKIDKTGRIFQFSEKPN |
| MaAPL2c | ILSGDQLYRMDYMDLVQKHVDTGADITVSCVPVGHSRASDYGLVKIDEAGRIIQFFEKPK |
| MaAPL3 | ILSGDQLYRMDYMDFVQRHIDTGADITISCVPVSSSRASDYGIVKIDKAGHIIQFSEKPK |
| MaAPS1 | ILAGDHLYRMDYEKFIQAHRETNADITVAALPMDEKRATAFGLMKIDEEGRIIEFAEKPK |
| MaAPS2 | VLPGHHLYEMDYRKLIKAHRDNRADITIAMANNDRNYDASHDFLLHTSKNQFHGLMLAPA |
| | .* * .** *** .. * ****.. . . .. * |
| MaAPL1a | GEDLKAMEVDTSVLGLSKEEAAKNPYIASMGVYVFKKELLLNLLRWRFPTANDFGSEIIP |
| MaAPL1b | GGDLETMKDENTLFRLSHQDAKKYPYIASMGVYVFNRNTLLELLRWTYPKANDFGSDILP |
| MaAPL2a | GAELEAMKDGGSFLRLSRQDAMKYPYIASMGVYVFKRDVLSKLLRWKYPKANDFGSEILP |
| MaAPL2b | GAELEAMKDGGSFLRLSRQDAMKYPYIASMGVYVFKRDVLSKLLRWKYPKANDFGSEILP |
| MaAPL2c | GADLEAMNDNGTFLRLSHQDAMKYPYIASMGVYVFKRDVLLKLLRWNYPKCNDFGSEILP |
| MaAPL3 | GADLEAMKDENTFFRLSHQDTIRYPYIASMGVYVFNRNTLLELLRWTYPKANDFGLDILP |
| MaAPS1 | GDQLKAMKVDTTILGLDNERAKEMPFIASMGIYVISKDIMLQLLRDKFAGANDFGSEVIP |
| MaAPS2 | S-------VHSPSAAVTTTTKYPVSNAENMGIYVIRRDILIELLQEQLPKANDFGTEVLQ |
| | . **.** . . **. .**** ... |
| MaAPL1a | -ASAKEFFIKAYLFNDYWEDIGTIKSFFEANLALTA-----------------------H |
| MaAPL1b | -SAVKDYNVQAYIFKDYWEDIQTIKSFYDANLALTD----------------------Q |

| | |
|---|---|
| MaAPL2a | -SVVKEHNVQAYIFNDYWEDIGTIRSFFDANLALTEQVGISFRRTHANFMLKISSELFVQ |
| MaAPL2b | -SVVKEHNVQAYIFNDYWEDIGTIRSFFDANLALTE-----------------------Q |
| MaAPL2c | -SAVEEHNVQAYAFSDYWEDIGTIRSFFDANLALTE-----------------------Q |
| MaAPL3 | -SAVKAYKAQAYIFEDYWDDIGTIKSFYDANLALTE-----------------------Q |
| MaAPS1 | GATNVGMRVQAYLYDGYWEDIGTIEAFYNANLGITKK----------------------P |
| MaAPS2 | GAIALGMKVHAYMFDGHWNDLRNIEAFYQVNIESIT-----------------------R |
| | .       .** .   * *. .* .*.  *. |
| MaAPL1a | PPRFSFYDATKPTYTSRRNLPPSKVDNSKIVDSIVSHGTFMDNCLIEHSVIGIRSRISSN |
| MaAPL1b | PPKFQFYDPRTPIFTSPRFLPPNKIEQCRIRDAIISHGCFLHECSIEHSVVGMRSRVYYG |
| MaAPL2a | PPKFQFYDPMTPFFTSPRFLPPTKIEKCRIVDAIISHGCFLRECSVERSIVGVRSRLDFD |
| MaAPL2b | PPKFQFYDPMTPFFTSPRFLPPTKIEKCRIVDAIISHGCFLRECSVERSIVGVRSRLDFD |
| MaAPL2c | PPKFQFYDPMTPFFTSPRFLPPTKIEKCRVMDAIISHGCFLRECSVERSIVGVRSRLDFG |
| MaAPL3 | PPKFQFYDPRTPIFTSPRFLPPTKIEKCRILDSIISHGCFLHECSIEHSIVGVRSRIDYG |
| MaAPS1 | VPDFSFYDRTSPIYTQPRYLPPSKMLDADVTDSVIGEGCVIKNCKIHHSVIGLRSCISEG |
| MaAPS2 | SMSTNFHGRHPAIYTLPNYLPPTTISNALIKDSIIGDGCLLNRCKISNSVIGTRTYVGDD |
| |      *       .*    ***  .  . . *...  *. .  * .  *..* *. . |
| MaAPL1a | VHLKDTVMLGADYYETDAETAS-LLAEGRVPIGIGENTKIRNCIIDKNARIGKNVIISNS |
| MaAPL1b | AELKHTLMMGADDYETEAEIAS-LLAEGKVPIGVGENTKIRNCIIDMNARIGKNVVIANR |
| MaAPL2a | AELKDTMMMGADIYETEAEIAS-LLADDKVPIGVGQNTRIRNCIIDMNARIGKNVVIANK |
| MaAPL2b | AELKDTMMMGADIYETEAEIAS-LLADDKVPIGVGQNTRIRNCIIDMNARIGKNVVIANK |
| MaAPL2c | VELKDTMMMGADIYETEAEISS-HLADDKVPIGVGQKTKIRNCVIDMNARIGKNVVIANK |
| MaAPL3 | AELKDALMLGADLYETEAELAS-ILAEGKVPIGVGQNTKIRNCIIDMNARIGKNVVIANK |
| MaAPS1 | AVLEDTLLMGADYYETDADRRL-LAAKGSVPMGIGRNSHVKRAIIDKNARIGENVKIINC |
| MaAPS2 | AVIKKSVVMGSDIYEADVSWNTTKMQRSEIPVGIGEKAHVQNAIIDKNARIGKNVKIVNK |
| |   .  ....*.* **..            .*.*.*  .... ..** ***** ** * * |
| MaAPL1a | DGIQEADRSAEGFYIRSGVTIILKNSTIGDGFVI |
| MaAPL1b | DGVQEADRPSEGFYIRSGITTILKSATVKDGTVI |
| MaAPL2a | DGIQEADRPCEGFYIRSGITIIMKNSTIKDGTVI |
| MaAPL2b | DGIQEADRPCEGFYIRSGITIIMKNSTIKDGTVI |
| MaAPL2c | DGVQEADRASEGFYVRSGIVVILKNATIKDGTVI |
| MaAPL3 | DGVQEADRPSEGFYIRFGITIIMKNATIKDGTVI |
| MaAPS1 | DNVQEAARETDGYFIKSGIVTVIKDALIPSGTVI |
| MaAPS2 | DGVQECDREASGYIISGGIVVVLKNAVIPDDSIL |
| | *..**. * . *. .  *.  ..* . .    .. |

**Figure S1.**
